# Comparative population genetics and evolutionary history of two commonly misidentified billfishes of management and conservation concern

**DOI:** 10.1186/s12863-014-0141-4

**Published:** 2014-12-14

**Authors:** Andrea M Bernard, Mahmood S Shivji, Eric D Prince, Fabio HV Hazin, Freddy Arocha, Andres Domingo, Kevin A Feldheim

**Affiliations:** The Guy Harvey Research Institute, Oceanographic Center, Nova Southeastern University, 8000 N. Ocean Drive, Dania Beach, FL 33004 USA; National Marine Fisheries Service, Southeast Fisheries Science Center, 75 Virginia Beach Drive, Miami, FL 33149 USA; Departamento de Pesca e Aquicultura, Universidade Federal Rural de Pernambuco, Rua Dom Manoel de Medeiros, s/n, Dois Irmãos, Recife, PE 52171-032 Brazil; Instituto Oceanográfico de Venezuela, Universidad de Oriente, Apartado de Correos, 204, Cumaná, 6101 Venezuela; Laboratorio de Recursos Pelágicos, Dirección Nacional de Recursos Acuáticos, Constituyente 1497, Montevideo, CP 11200 Uruguay; The Field Museum of Natural History, Pritzker Laboratory for Molecular Systematics and Evolution, 1400 South Lake Shore Drive, Chicago, IL 60605 USA

**Keywords:** Roundscale spearfish, White marlin, Genetic population structure, Genetic diversity, Effective population size, *Tetrapturus georgii*, *Kajikia albida*

## Abstract

**Background:**

Misidentifications between exploited species may lead to inaccuracies in population assessments, with potentially irreversible conservation ramifications if overexploitation of either species is occurring. A notable showcase is provided by the realization that the roundscale spearfish (*Tetrapturus georgii*), a recently validated species, has been historically misidentified as the morphologically very similar and severely overfished white marlin (*Kajikia albida*) (IUCN listing: Vulnerable). In effect, no information exists on the population status and evolutionary history of the enigmatic roundscale spearfish, a large, highly vagile and broadly distributed pelagic species. We provide the first population genetic evaluation of the roundscale spearfish, utilizing nuclear microsatellite and mitochondrial DNA sequence markers. Furthermore, we re-evaluated existing white marlin mitochondrial genetic data and present our findings in a comparative context to the roundscale spearfish.

**Results:**

Microsatellite and mitochondrial (control region) DNA markers provided mixed evidence for roundscale spearfish population differentiation between the western north and south Atlantic regions, depending on marker-statistical analysis combination used. Mitochondrial DNA analyses provided strong signals of historical population growth for both white marlin and roundscale spearfish, but higher genetic diversity and effective female population size (1.5-1.9X) for white marlin.

**Conclusions:**

The equivocal indications of roundscale spearfish population structure, combined with a smaller effective female population size compared to the white marlin, already a species of concern, suggests that a species-specific and precautionary management strategy recognizing two management units is prudent for this newly validated billfish.

**Electronic supplementary material:**

The online version of this article (doi:10.1186/s12863-014-0141-4) contains supplementary material, which is available to authorized users.

## Background

Identifying genetic conservation units of large-bodied, marine pelagic fishes remains challenging as a result of their often large population sizes, typically strong dispersal ability (via adult and/or larval phases) and few apparent physical barriers to gene flow. These parameters are generally associated with shallow levels of genetic differentiation across large geographic regions [1-3]. More recently, however, low but statistically significant levels of genetic differentiation have been detected among populations of pelagic fishes, introducing exceptions to the traditional paradigm of little if any genetic structure across local and even broad spatial scales for such taxa [4,5]. Although the biological interpretation of such shallow genetic differentiation is sometimes unclear [2,6], defining genetic population boundaries remains essential for conservation of genetic legacies and adaptive potential. This issue is of particular interest in the case of apex predatory fishes given their likely important ecosystem role, and the fact that many are also exploited in highly valuable commercial and recreational fisheries. Istiophorid (Istiphoridae) billfish species fall in this category, and in several cases are known to have declined to levels low enough to cause international concern about their population status [7-10].

**Figure 1:**
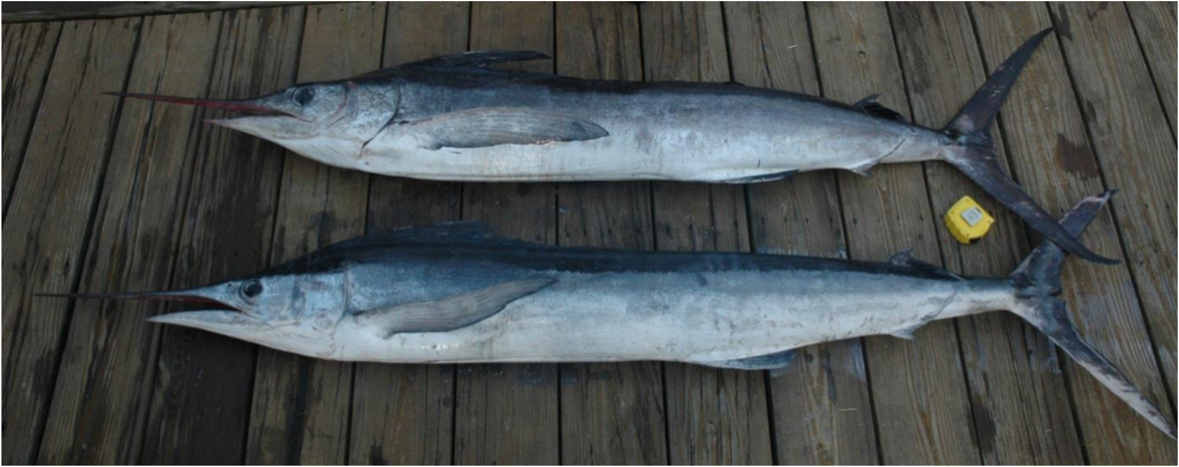
**White marlin (top) and roundscale spearfish (bottom) showing strong morphological similarity.** (Image credit: J. Foster/GHRI)

The recent validation of the roundscale spearfish (*Tetrapturus georgii*) and its routine misidentification as the morphologically very similar and overfished, sympatric white marlin, *Kajikia albida* (Figure 1), have raised new management and conservation challenges concerning the population status of both species [11-14]. While genetic analyses can readily differentiate these two species [11,15,16], only subtle morphological differences distinguish them, requiring either very close visual examination or the taking of morphometric measurements. These differences are: visual - shape of lateral torso scales; morphometric - ratio of the distance from the anus to the origin of the first anal fin to the maximum height of this fin, and branchiostegal to opercle length relationship [17]. Further exacerbating these challenges is that the roundscale spearfish and white marlin possess largely sympatric Atlantic-wide temperate and tropical distributions [13], and that misidentifications may also be occurring between these species and the longbill spearfish (*Tetrapturus pfluegeri*), a third morphologically similar species [7].

Two intertwined issues have complicated assessing the population status and planning of management strategies for these billfishes: (i) the widespread species misidentifications in the context of severe white marlin declines, and (ii) the current lack of almost any data for the roundscale spearfish. First, white marlin have undergone severe population declines over the past four decades, mostly as a result of offshore longline fisheries in the Atlantic [10]. This species is currently listed as “Vulnerable” on the IUCN Red List of Threatened Species [8]. In addition, it is now recognized that decades of unrealized species misidentifications have occurred between the white marlin, and the longbill and roundscale spearfish, and that management strategies for the nominal “white marlin” have unknowingly been based on catch information and stock assessments for a species-complex [7,18]. Potential impacts of these species misidentifications may be severe [10]. Analysis of commercial catch data has suggested that the roundscale spearfish may comprise a significant proportion (27%) of the overall “white marlin” catch in the western North Atlantic and that both species may show contemporary evidence of over-exploitation and decline [18]. Further hindering management efforts, is the second relevant issue that almost nothing is known about the population dynamics of the roundscale spearfish, including its population genetic structure and demographic history.

Here, we provide the first population genetic assessment of the roundscale spearfish to inform management and conservation efforts for this enigmatic, recently recognized species. As part of this assessment, we utilize nuclear microsatellite and mitochondrial sequence markers to explore the population structure of this species in its western Atlantic range. Furthermore, we utilized existing white marlin mitochondrial DNA sequences to compare the genetic diversity and evolutionary history of the white marlin and roundscale spearfish, as the bulk of misidentifications are believed to occur between these two species [11,18]. As historical stock assessments of the white marlin were based on landings that were unknowingly comprised of a species-complex, this comparison allowed for a unique species-specific survey of the demographic history of two morphologically very similar, but evolutionarily distinct species.

## Methods

### Ethics statement

The roundscale spearfish tissue samples used in this study were obtained from fish harvested independently by commercial fisheries. This is not a CITES listed species and no permits or licenses were required to work with these samples. All laboratory work on these samples was performed in accordance with Nova Southeastern University guidelines.

### Samples and collection sites

**Figure 2:**
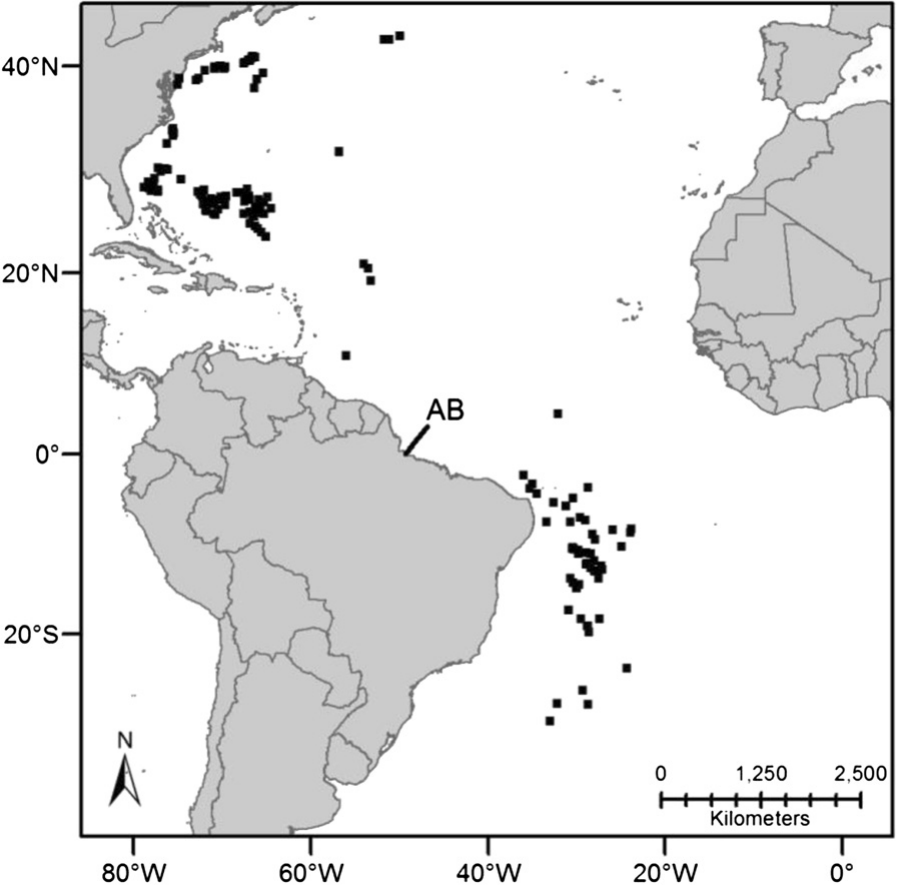
**Map of sampling distribution of roundscale spearfish (** ***Tetrapturus georgii*** **); (**_▀_ **) represents the capture location of a single individual; (AB) represents the location of the Amazon River Biogeographic Barrier.**

A total of 198 roundscale spearfish samples were obtained from animals incidentally caught in long-line fisheries targeting other teleost species, including swordfish and tuna. The majority of individuals possessed a lower jaw fork length ranging from 126 to 186 cm (where data was available). To date, no information is available on the relationship between length and maturity for roundscale spearfish; however, assuming size at maturity of the roundscale spearfish and white marlin are comparable [19,20], our roundscale spearfish comprised mainly adult fish. Roundscale spearfish capture locations are shown in Figure 2, and comprise locations within the western Atlantic, both north and south of the equator [i.e., the western North Atlantic (WNA; n = 140) and western South Atlantic (WSA; n = 58)]. Samples were divided into *a priori* western North and South populations for population-level analyses for two reasons: (i) presence of the Amazon plume at equatorial latitudes, a known biogeographic barrier for numerous reef fishes [21,22], and (ii) previous genetic evidence of at minimum weak genetic differentiation between North and South collections of other billfishes as well as the presence of disjunct hemispheric spawning locations for some billfishes [23,24].

Tissue samples were stored in 95% ethanol until genomic DNA extraction. Prior to inclusion in this study, identities of all roundscale spearfish were verified using a multiplex species-specific primer test targeting the mitochondrial protein coding gene NADH dehydrogenase 4 (MS Shivji unpublished observation). Identities of a subset of samples (n = 43) were also confirmed by mitochondrial Cytochrome c oxidase I barcodes [16]. To investigate the genetic population characteristics of the roundscale spearfish, we genotyped 198 individuals at 13 microsatellite loci, and sequenced ~580 base pairs (bp) of the mitochondrial control region (mtCR) locus of a subset of individuals (total n = 83: WNA n = 42; WSA n = 41). To assess the comparative genetic diversity and demographic history of the white marlin relative to roundscale spearfish, white marlin mtCR sequences (834 bp) from 99 individuals (91 haplotypes) were obtained from GenBank (Accession numbers DQ835191-DQ835281) comprising individuals sampled from the WSA, WNA, Caribbean Sea and the Eastern Atlantic [23]. As we only obtained partial mtCR locus sequences from roundscale spearfish, all white marlin sequences were cropped (to 601–605 bp) to ensure that the same region was analyzed for both species. Variation in length of mtCR sequences between species resulted from indels.

### Mitochondrial DNA sequencing and microsatellite genotyping

Genomic DNA was extracted from ~25 mg of roundscale spearfish tissue using the DNeasy Kit (QIAGEN Inc., Valencia, CA) following manufacturer’s instructions. To amplify and sequence the ~580 bp section of the mtCR, we used the primer pair Pro-5M13F (5′-CAC GAC GTT GTA AAA CGA CCT ACC YCY AAC TCC CAA AGC-3′) and dLoopi (5′-CCA TCT TAA CAT CTT CAG TG-3′) [15]. Total polymerase chain reaction (PCR) volumes were 50 μL and contained 1 μL of unquantified extracted genomic DNA. Final concentration of the remaining PCR reactants were 1 x PCR buffer (0.15 mM MgCl_2_), 0.2 mM of each dNTP, 0.25 μM of each of the Forward and Reverse primers and 1.0 U of HotStar *Taq*™ DNA Polymerase (QIAGEN Inc.). PCR was performed in a Mastercycler Gradient (Eppendorf Inc., Westbury, NY) thermal cycler as follows: an initial denaturation at 95°C for 15 minutes (min), followed by 35 cycles of 94°C for a 1 min, 50°C for 1 min, 72°C for 1 min, and a 20 min final extension step at 72°C. A negative control (no genomic DNA) was included in each PCR set to check for reagent contamination. PCR products were purified using the QIAquick PCR Purification Kit (QIAGEN Inc.) and double-strand sequenced using standard protocols on an AB 3130 genetic analyzer (Applied Biosystems Inc., Foster City, CA). The mtCR sequences were aligned using MUSCLE as implemented in the program Geneious version 6.0.6 (Biomatters Inc., San Francisco, CA), and the alignment was subsequently refined and manually checked by hand.

The 13 microsatellite loci used for genotyping were those developed for roundscale spearfish by Bernard et al*.* [25] (*tge*23, *tge*54, *tge*76, *tge*79, *tge*105, *tge*119, *tge*135, *tge*139, *tge*144, and *tge*151) and blue marlin (*Makaira nigricans*) by Buonaccorsi and Graves [26] (Mn01, Mn10, and Mn60). The roundscale spearfish species-specific microsatellite loci were amplified as per Bernard et al. [25]. The three blue marlin microsatellite loci, were cross-amplified in roundscale spearfish using a total PCR reaction volume of 25 μL, containing 1 μL (unquantified) genomic DNA. Final concentration of the remaining PCR reactants were 1 x PCR buffer (0.15 mM MgCl_2_), 0.2 mM of each dNTP, 0.33 mM MgCl_2_, 0.16 μM of the Forward microsatellite primer which possessed a 5′-M13 tail [27], 0.4 μM of the Reverse microsatellite primer, 0.4 μM of the fluorescently labeled universal M13 primer (5′-TGTAAAACGACGGCCAGT-3′) [27], and 0.5 U of HotStar *Taq*™ DNA Polymerase (QIAGEN Inc.). PCR was performed in a Mastercycler Gradient (Eppendorf Inc.) thermal cycler as follows: 95°C initial heating for 15 min, followed by 35 cycles of 94°C for 1 min, 1 min at the primer annealing temperature [*T*_A_ = 60°C (Mn01, Mn10, Mn60, *tge*105, *tge*119, *tge*135, *tge*139, and *tge*151), and 58°C (*tge*23, *tge*54, *tge*76, *tge*79, *tge*144)], 72°C for 1 min, and a final 20 min extension step at 72°C. Electrophoresis was performed on an AB 3130 (Applied Biosystems Inc.) genetic analyzer. All fragments were sized using LIZ 600 as the internal allele size standard and scored using the software GENEMAPPER 3.7 (Applied Biosystems Inc.).

### Data analysis

Calculations of microsatellite allele frequencies, expected (*H*_E_) and observed (*H*_O_) heterozygosities, and tests for Hardy-Weinberg (HWE) and linkage equilibrium (LE) were performed using GENEPOP on the web (v.4.0.10) [28,29]. To estimate the significance of the above tests, we used an unbiased exact test, employing the Markov chain method (1000 dememorizations, 100 batches, 1000 iterations per batch) [30,31] as implemented in GENEPOP. Significance levels were adjusted using sequential Bonferroni correction [32] to accommodate multiple comparison testing. Microsatellite allelic richness (*R*_S_) [33] for each collection site (*a priori* defined as samples from the WNA or WSA) was estimated using FSTAT 2.9.3.2 [34]. The frequency of null alleles was estimated using the program FreeNA [35].

### Population genetic structure of roundscale spearfish: population-level analyses

To test for western Atlantic population subdivision with both mitochondrial and nuclear markers, we estimated divergence between WNA versus WSA samples. For mtCR sequence data, divergence was estimated using Ф_ST_ [Tamura and Nei (TN) model of evolution; 10 000 permutations] as implemented in Arlequin 3.1 [36], Jost’s *D* statistic [37] as implemented in the program SPADE [38] (10 000 bootstrap iterations), and the nearest neighbor statistic (*S*_nn_) [39] as implemented in DnaSP v5 [40] [significance of the *S*_nn_ test statistic was estimated using 10 000 permutations (sites with alignment gaps excluded)].

For microsatellite data, between population divergence was estimated using Jost’s *D* (arithmetic mean of *D*_est_) using DEMEtics [41] within the statistical package R v2.15.1 [42] (significance estimated using 1000 bootstrap iterations), and *F*_ST_ as implemented in FSTAT. Gender information was available for a large sub-set of our roundscale spearfish samples (uncommon in billfish landings data), as such we tested for sex-biased dispersal (FSTAT: 1000 randomizations). Although sex-biased dispersal has not been documented previously in istiophorid billfish, Muths et al*.* [43] found support for this hypothesis in swordfish (*Xiphias gladius*), raising the potential for sex-biased dispersal in other migratory billfishes. All seven measures were utilized to test for sex-biased dispersal.

### Population genetic structure of roundscale spearfish: individual-level analyses

The Bayesian multi-locus clustering program *Structure* v2.31 [44] was utilized to determine the most likely number of genetically discrete populations [Ln Pr (*X*|*K*)]. Two disparate *Structure* analyses (see below) were performed both consisting of ten replicates for the values *K* = 1 - 5 (MCMC chain length and burn-in consisted of 200 000 and 100 000 iterations, respectively), assuming correlated allele frequencies [45] and admixture. One analysis was performed without *a priori* sampling location information, while the second analysis implemented the model *locprior* [46], which incorporates *a priori* sampling location information (e.g., WNA versus WSA in this case).

Potential genetic spatial discontinuities were also assessed using the program Geneland 3.1.4 [47] as implemented in the statistical package R [42] to complement *Structure*’s individual-based analyses. All runs incorporated the Dirichlet distribution model of independent allele frequencies [47]. Geneland was run 10 times at *K* = 1 - 5 for 500 000 iterations (500 thinning; 50 000 burn-in) with zero uncertainty of geographical coordinates. Given the highly migratory nature of roundscale spearfish, additional Geneland analyses were performed assuming varying levels of coordinate uncertainty (10 km and 100 km). Geographic coordinates used represented the location of the start of the fishing long-line set on which each individual roundscale spearfish was captured.

To evaluate the hypothesis of whether genetic distance among roundscale spearfish individuals was correlated with geographical distance among their collection locations, Mantel tests were performed as implemented in GenAlEx [48]. The significance of correlations was assessed using 999 permutations.

### Comparative mitochondrial DNA-based genetic diversity and demographic histories of the roundscale spearfish and white marlin

We used the software jModelTest 2.1.2 [49,50] to identify the most appropriate model of DNA evolution using the Akaike information criterion (AIC) for both mtCR sequence datasets. Molecular diversity calculations for mtCR sequences (number of unique haplotypes, number of segregating sites, nucleotide composition, and haplotype (*h*) and nucleotide diversities (π) estimated with Nei’s corrected average genetic divergence [51]) were estimated in Arlequin.

To test for departures from a constant population size we estimated the summary statistics Fu’s *F*_S_ [52] in Arlequin (10 000 iterations) and *R*_*2*_ [53] in DNAsp. Significance of the *R*_2_ statistic was determined in DNAsp using 10 000 replicates. We note that 5 of the 91 white marlin mtCR sequences downloaded from NCBI (Accession numbers DQ835236, DQ835248, DQ835251, DQ835275, and DQ835277) contained ambiguous bases. Since DNAsp does not allow for ambiguous bases, these five sequences were not included in the *R*_2_ analysis.

Demographic expansions were also assessed for both species by mismatch analyses [54] using the sudden demographic expansion model implemented in Arlequin. Model fit to our data was statistically tested using the sum of squared deviations (SSD) and the raggedness index (*Hri*) [55] (1000 bootstrap replicates). Mismatch analyses were performed for each *a priori* roundscale spearfish population as well as for the overall (pooled) roundscale spearfish mitochondrial dataset. Population parameters τ, Θ_0_, and Θ_1_, where Θ_0_ and Θ_1_ are the expected pairwise differences before and after a change in population size, respectively [55], and τ is a relative measure of time since population expansion in generations, were also estimated. Using the above parameters, actual time since population expansion (*t*) was estimated as t = τ/2μ, where μ is the mutation rate per locus per generation. Reported estimates of teleost mitochondrial control region divergence rates have varied considerably across species and studies, but typically range between 3.6 – 9% per site per million years [56-58]. Given the absence of specific divergence rates for the istiophorid lineages, we adopted the provisional mutation rates of 1.8 – 4.5% per site per million years based on the typical divergence rates reported (note: mutation rate within a lineage = ½ divergence time between lineages) [56,57], to determine time since population expansion from the mismatch distribution for the two billfishes. We estimated the long-term population parameters Θ (2*Nf*_E_μ, where *Nf*_E_ = the female historical effective population size, and μ = the mutation rate) and exponential growth rate (*g*) for roundscale spearfish and white marlin using the pooled sample set for each species, as well as separately for each of the WNA and WSA roundscale spearfish sample sets using the Bayesian method implemented in the program LAMARC 2.0 [59]. Assuming that the roundscale spearfish and white marlin share similar mtCR mutation rates, Θ will allow for a direct comparison of each species’ relative female effective population size and the relative genetic variability found within species [60]. Analyses were performed using the GTR and F84 models of evolution (roundscale and white marlin samples, respectively) and three simultaneous chains implementing an adaptive heating scheme (1.0, 1.1, 1.3). A single chain comprising 10 to 20 million iterations was employed (10% burn-in). Convergence was assessed using the program Tracer 1.5 [61]; estimates were assumed to have converged once ESS scores exceeded 200 for all parameter estimates. Final parameter estimates and credibility intervals were the most probable estimates (MPE) determined after three replicates.

To assess the historical trajectories in female effective population size for roundscale spearfish and white marlin, we constructed coalescent-based Bayesian skyline plots (BSP) using the program BEAST v1.5.2 [62]. Plots were constructed for each *a priori* population of roundscale spearfish (i.e. WNA and WSA) and for pooled samples from each species. Priors included the implementation of the TVM + I + G and HKY + I + G models of substitution for roundscale spearfish and white marlin datasets, respectively (as defined by jModelTest), and the strict clock model. Priors for the site heterogeneity model [Gamma (G) and Invariant Sites (I)] were obtained from jModelTest. The piecewise-constant skyline model was selected and runs were fixed at 10 groups. The fixed substitution rate was set to correspond to the mutation rates utilized in the previous analyses (1.8 – 4.5% per site per million years). MCMC tests were run for 50 million generations and sampled every 5000^th^ step (10% burn-in). Convergence was assessed using the program Tracer and estimates were assumed to have converged once ESS scores exceeded 200 for all parameter estimates.

## Results

### Population genetic structure of roundscale spearfish: population-level analyses

The genotypes of 198 roundscale spearfish were determined at 13 microsatellite loci. Sample sizes, basic genetic diversity statistics and the deviation from HWE for each locus and across all loci for each sampling location are listed in Table 1. Average heterozygosities and allelic richness for both roundscale spearfish populations across all loci ranged from 0.71-0.74 and 14.3-14.6, respectively. All loci met HWE expectations after sequential Bonferroni correction (α/26; α = 0.05); however, pairwise tests of LE within populations demonstrated significant disequilibrium between three locus pairs (WNA: Mn01 & *tge*105, *tge*23 & *tge*54; WSA: *tge*135 & *tge*139) after sequential Bonferroni correction (*P* < 0.05). We note, however, that where significant departures from LE were detected; they were not widespread, being restricted to only one of the two *a priori* defined populations (i.e., WNA or WSA). Furthermore, no evidence of linkage disequilibrium (LD) was found when all roundscale spearfish samples were pooled, suggesting that the surveyed microsatellite loci do sort independently. The frequency of null alleles estimated by FreeNA across all genotyped loci was < 5.0% and therefore considered negligible for analysis purposes [35,63].

**Table 1:** **Summary statistics of 13 microsatellite loci for roundscale spearfish (** ***Tetrapturus georgii*** **)**

	**Locus**													**Average across loci**
**Sample**	**MN01**	**MN10**	**MN60**	***tge*** **23**	***tge*** **54**	***tge*** **76**	***tge*** **79**	***tge*** **105**	***tge*** **119**	***tge*** **135**	***tge*** **139**	***tge*** **144**	***tge*** **151**	
**WNA**														
*n*	137	137	127	140	135	139	139	139	134	135	136	134	128	--
*a*	18	27	32	4	14	3	17	19	28	24	10	23	8	17.5
*R*_S_	15.8	21.8	25.2	3.6	12.3	2.7	14.1	14.6	21.0	22.0	8.3	18.2	6.7	14.3
*as*	267-335	291-439	217-319	223-235	202-232	103-109	129-197	185-227	187-238	177-277	99- 139	110-212	191-205	--
*H*_O_	0.90	0.94	0.92	0.44	0.86	0.05	0.78	0.73	0.87	0.89	0.76	0.77	0.63	0.73
*H*_E_	0.90	0.93	0.94	0.50	0.86	0.06	0.78	0.73	0.89	0.94	0.71	0.82	0.63	0.74
*HWE*	0.02	0.67	0.47	0.25	0.01	0.09	0.80	0.75	0.44	0.05	0.55	0.01	0.11	0.002
**WSA**														
*n*	58	55	57	58	58	57	58	58	55	56	57	57	58	--
*a*	16	22	30	4	12	3	13	12	25	21	7	19	7	14.7
*R*_S_	15.8	22	29.6	4.0	11.8	3.0	12.8	11.9	25	20.9	6.9	18.8	6.9	14.6
*as*	275-335	291-427	193-331	211-231	200-226	103-109	129-183	193-225	185-229	177-277	99- 127	110-198	189-207	--
*H*_O_	0.95	0.95	0.91	0.47	0.76	0.07	0.72	0.74	0.87	0.95	0.74	0.70	0.45	0.71
*H*_E_	0.91	0.93	0.95	0.43	0.84	0.07	0.79	0.72	0.90	0.81	0.72	0.74	0.53	0.72
*HWE*	0.86	0.05	0.24	0.57	0.64	1.00	0.26	0.92	0.22	0.99	0.11	0.67	0.08	0.402

*Abbreviations:* *WNA* western North Atlantic, *WSA* western South Atlantic, *n* number of individuals, *a* number of alleles, *R*_S_ allelic richness, *as* size range of alleles, *H*_O_ observed heterozygosity, *H*_E_ expected heterozygosity, *HWE* probability of conformation to Hardy-Weinberg expectations.

Due to the significant LD between some loci, we utilized three post-hoc pairwise estimates of *F*_ST_ to assess population differentiation between WNA and WSA roundscale spearfish: fixation indices were computed for 1) each locus individually, 2) across all 13 loci, and 3) across just 10 loci (i.e., excluding loci Mn01, *tge*54, and *tge*139 to eliminate any disequilibrium bias). Individual locus *F*_ST_ estimates of divergence between WNA and WSA roundscale spearfish populations ranged between −0.0046 to 0.0194, with five of 13 loci providing significant estimates of divergence at *P* < 0.05 (Mn1, Mn10, *tge*119, *tge*144, *tge*151). Across all 13 microsatellite loci, the overall *F*_ST_ was estimated at 0.0037 and was significant at *P* = 0.05. The 10 locus *F*_ST_ estimate was also 0.0037 and significant (*P* = 0.05). Estimates of the arithmetic mean of the *D*_est_ statistic were low but statistically significant: *D*_est_ = 0.021 (*P* = 0.005) and 0.019 (*P* = 0.012) for the 13 and 10 loci, respectively. No statistically significant, nuclear marker based evidence of sex-biased dispersal was detected in any of the parameters estimated across either suite of 10 or 13 loci.

Mitochondrial DNA analyses provided mixed evidence of differentiation between the WNA and WSA roundscale spearfish. The Ф_ST_ estimate of 0.0046 was non-significant (*P* = 0.24); in contrast, the S_nn_ statistic was 0.625 and significant (*P* = 0.017). Jost’s *D* test statistic showed no divergence as *D* was estimated at −0.061 (95% confidence intervals 0.000, 0.146).

### Population genetic structure of roundscale spearfish: individual-level analyses

Results from the 13- and 10-locus microsatellite data sets were congruent for all individual-based analyses. *Structure* identified a single, homogenous population of roundscale spearfish within western Atlantic waters. Mean Ln Pr (*X*|*K*) values across the ten runs peaked at *K* = 1 for both model-type analyses [without spatial model: Mean Ln Pr (X|K) = −10177.3; with *locprior* model: Mean Ln Pr (X|K) = −10177.3], and variances associated with likelihood estimates increased at *K* > 1 (not shown). Geneland derived posterior distributions of the estimated number of populations (*K*) also produced a clear mode at *K* = 1 for all 10 runs. Log likelihoods ranged from −8805 (run 4) to −9002 (run 9) and no evidence of genetic subdivision among samples was detected (not shown). Coordinate uncertainty had no effect on the estimated number of populations (not shown). Individual-based Mantel tests revealed a lack of significant correlation between pairwise genetic and geographical distance among individuals for all comparisons (*R*^2^ = 0.00001; *P* = 0.487).

### Comparative mitochondrial DNA-based genetic diversity and demographic histories of the roundscale spearfish and white marlin

Sample sizes and population-level mitochondrial diversity indices for both billfish species are listed in Table 2. Sequencing 577–580 base pairs (bp) of the roundscale spearfish (total n = 83) mtCR resolved 69 haplotypes consisting of 17.83% cytosine, 32.70% thymine, 34.74% adenine, and 14.74% guanine (GenBank Accession no. KF441482-KF441550). The sequences revealed 154 polymorphic sites consisting of 134 transitions, 19 transversions, and 15 indels. Overall haplotype (*h*) and nucleotide (π) diversities were 0.993 ± 0.004 and 0.024 ± 0.012, respectively, and were similar between the WNA and WSA samples. In comparison, white marlin mtCR sequences (n = 99) resolved 91 haplotypes consisting of 21.69% cytosine, 28.84% thymine, 30.48% adenine, and 18.99% guanine. A total of 225 polymorphic sites were identified, consisting of 208 transitions, 10 transversions, and 22 indels. Overall, mtCR genetic diversity estimates in white marlin were higher than roundscale spearfish (Table 2, see *h*, *π*, and Θ**).**

**Table 2:** **Mitochondrial control region sequence variability and population demographic parameters for roundscale spearfish and white marlin**

**Species and Sample**	***n***	***nh***	***h***	**π**	**MPE Θ (95% CI)**	**MPE** ***g*** **(95% CI)**	***F***_S_	***R***_2_
RS WNA	42	36	0.992 ± 0.007	0.024 ± 0.012	0.391 (0.206, 0.895)	220.04 (129.93, 352.30)	−17.00^†^	0.053^*^
RS WSA	41	37	0.994 ± 0.007	0.024 ± 0.012	0.219 (0.124, 0.447)	148.35 (68.13, 256.15)	−20.49^†^	0.058^*^
RS overall	83	69	0.993 ± 0.004	0.024 ± 0.012	0.402 (0.267, 0.666)	193.47 (125.30, 284.18)	−24.21^†^	0.044^*^
WM overall	99	91	0.998 ± 0.002	0.037 ± 0.018	0.772 (0.534, 1.226)	180.00 (130.55, 242.07)	−23.95^†^	0.045^*^

Abbreviations: RS, roundscale spearfish; WM, white marlin; WNA, western North Atlantic; WSA, western South Atlantic; *n*, number of individuals; *nh*, number of haplotypes; *h*, haplotype diversity, π, nucleotide diversity; MPE Θ, most probable estimate of Kuhner’s (2006) parameter theta; (95% CI), 95% credibility intervals; MPE *g*, most probable estimate of Kuhner’s (2006) exponential growth rate; *F*_S_, Fu’s (1996) test statistic; *R*_2_, Ramos and Rozas (2002) test statistic. ^*^indicates significance at *P* < 0.01; ^†^indicates significance at *P* < 0.001.

The TVM model of substitution plus invariable sites (*I*) and a gamma distribution (Γ) of rate heterogeneity across variable sites provided the best fit to the roundscale spearfish mtCR data set (jModelTest). The estimated parameters under this model were Γ = 1.1990, and *I* = 0.51. For white marlin, jModelTest identified the HKY + I + G model [64] (Γ = 1.0830, and *I* = 0.3240) as the most appropriate substitution model for the mtCR dataset. All demographic analysis results for roundscale spearfish and most (see below) for white marlin were consistent with a scenario of population expansions for both species. Estimates of Fu’s *F*_S_ were negative and significantly different from zero, whereas *R*_2_ values were small, positive, and statistically significant (Table 2) for all three roundscale spearfish collections and for the pooled white marlin collection. Furthermore, for both species, mismatch analyses revealed large differences in Θ_0_ and Θ_1_, also indicative of rapid population expansions (Suppl. online Additional file 1). Similarly, the mismatch distribution model fit statistics, Harpending’s [55] raggedness index and SSD, failed to differ significantly from that expected under a model of sudden population expansion (Suppl. online Additional file 1). The mismatch distribution for the pooled roundscale spearfish samples appeared smooth and unimodal, consistent with a model of population expansion. In contrast, the mismatch distribution for white marlin was distinctly bimodal indicative of a largely stable population size (Suppl. online Additional file 2), but inconsistent with the demographic summary test statistics for this species. Estimates of time since expansion (τ) for both roundscale spearfish populations overlapped substantially, suggesting a similar timing of demographic events in the WNA and WSA regions (Suppl. online Additional file 1). The mean timing of this expansion was estimated as τ = 10.97 for the pooled roundscale spearfish samples, and likely occurred approximately 211 000 – 530 000 years before present (ybp), assuming mutation rates of 4.5% and 1.8% per site per million years, respectively. Assuming the same mutation rates, the range of expansion times for the white marlin pooled samples was 235 000 – 585 000 ybp.

MPEs of Θ generated by LAMARC showed variation between the two species (and the two roundscale spearfish collections) (Table 2). The white marlin median estimate of Θ was approximately 1.9 times larger than that for roundscale spearfish, although substantial overlap among credibility intervals was found. Estimates of *g* were similar for both species and strongly positive, indicating substantial historical growth (Table 2).

**Figure 3:**
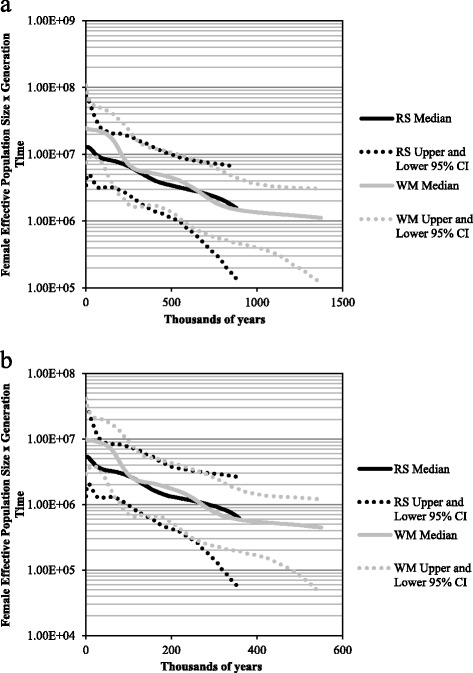
**Bayesian skyline plot (BSPs) estimated using BEAST from pooled samples of roundscale spearfish (RS:** ***Tetrapturus georgii*** **) and white marlin (WM:** ***Kajikia albida*** **); BSPs derived using a mutation rate of (a) 1.8% per site per million years, and (b) 4.5% per site per million years.**

Bayesian skyline plots provided a signal of mostly continuous historical population size growth for the roundscale spearfish and white marlin (Figure 3; Suppl. online Additional file 3). Credibility intervals (95%) around estimates of female *N*_E_ (female effective size x generation time) showed substantial overlap between species, although the final median estimates of female *N*_E_ for white marlin were roughly 1.5-1.9 times higher than roundscale spearfish.

## Discussion

### Roundscale spearfish population structure

We provide the first examination of the genetic population structure of the roundscale spearfish, a large, pelagic predator captured in international fisheries and whose existence has only recently been recognized. The mixed population structure inferences obtained from the different statistical approaches used here highlight some of the difficulties associated with identifying management units for pelagic teleosts with high vagility and contiguous distributions over large geographic scales. We address two issues relevant to deriving management and conservation inferences from our findings: (i) the discordance between population- and individual-level statistical analyses in the framework of the resolving power of these analyses, and (ii) the biological interpretation of the weak but significant genetic structure revealed by population-level statistical approaches.

Numerous studies have demonstrated that the use of highly polymorphic microsatellite markers in combination with population-level (pairwise) statistical tests have increased the ability to detect shallow genetic discontinuities between populations [65,66]. In contrast, individual, multilocus-based clustering or assignment methods may have lower power to resolve such weak genetic structure [66-68]. For example, rigorous testing of individual-based analyses suggests an inability to identify divergence below a threshold of *F*_ST_ < 0.01 - 0.03 [66-68]. While *F*_ST_ values below this magnitude often indicate low levels of genetic partitioning, biologically important differences between such mildly divergent populations may still be present, and should not be ignored as they may be relevant for the management and conservation of species of concern [69]. For roundscale spearfish, individual-based nuclear analyses (*Structure* and Geneland) failed to detect intra-specific genetic population structure between the northern and southern hemisphere sampling sites. In contrast, the significant *F*_ST_ (*P* = 0.05) obtained from population-level analyses supports the notion that at least shallow genetic differentiation exists between roundscale spearfish from the WNA and WSA. The individual-based analyses may not have resolved this shallow level of differentiation because it fell below their respective resolution thresholds.

Biological interpretation of the results of roundscale population-level analyses is complicated by the mixed outcomes obtained, which were dependent on the combination of marker and statistical test used. For example, even though population differentiation was not observed using individual-based analyses, the microsatellite pairwise statistical tests (*F*_ST_ and *D*_est_) were notably congruent in suggesting very shallow but statistically significant divergence between WNA and WSA roundscale spearfish collections. While some controversy exists surrounding the relative utility of the estimators *F*_ST_ and *D*_est_ when paired with highly variable microsatellite genetic markers [37,41,70], the fact that both estimators provided congruent results, support the inference that shallow genetic differentiation may exist between WNA and WSA populations.

Mitochondrial DNA analyses also revealed similar contradictions for inferences of roundscale spearfish population structure. Estimates of Ф_ST_ suggested an absence of differentiation, but the *S*_nn_ statistic identified significant differentiation among collections (*P* = 0.017). As roundscale spearfish haplotype diversity was quite high (*h* = 0.992 – 0.994), the *S*_nn_ test is likely a more powerful statistic than the traditional Ф_ST_ statistic to measure population-level differentiation [39].

Previous surveys of the genetic population structure of other istiophorid species (white marlin, blue marlin [*Makaira nigricans*] and sailfish, [*Istiophorus platypterus*]), have also found little, if any, support for population structure within the Atlantic [23,71-74]. However, notable parallels may be found when comparing the population structure of roundscale spearfish to the sympatric and also Atlantic-limited white marlin. For example, analysis of white marlin genetic population structure utilizing microsatellite markers and mtCR sequences also provided mixed inferences, depending on marker class and analysis method used. Previous work [23], employed five microsatellite loci and found a small but statistically significant *F*_ST_ of 0.0041 (*P* = 0.017) between WNA and WSA collections, which is very similar to the level of differentiation we found for roundscale spearfish (*F*_ST_ = 0.0037). Furthermore, as was found with the roundscale spearfish, the white marlin mtCR data did not detect significant differentiation between the WNA and WSA samples based on the Ф_ST_ statistic [23]. However, in contrast to the roundscale spearfish results, the *S*_nn_ statistic did not differentiate white marlin from the WNA and WSA, despite both species having similarly high haplotype diversities. These contrasting *S*_nn_ results may be due to the small white marlin sample sizes used for the mtCR analyses (n = 20 per collection), which likely reduced the power to detect shallow differentiation [23,75]. Based on the mixed results across divergent marker classes, along with the weak, albeit significant and nearly significant, spatial differentiation obtained with microsatellite markers (varied by analysis method), Graves and McDowell [23] recommended continued management of white marlin as a single, Atlantic-wide stock. However, given some indications of population heterogeneity, they also recommended that this issue be further investigated with more microsatellite markers, better planned sampling design and larger sample sizes.

Such shallow population differentiation in roundscale spearfish, despite sampling from geographically distant regions (northern *vs.* southern hemispheres), raises the question of whether these populations should be treated as separate management units (MUs, *sensu* [76]). While it is possible that such shallow differentiation is a result of sufficient gene flow occurring between hemispheres to prevent accumulation of larger genetic differences, it is also possible that fine scale demographic independence exists between roundscale spearfish from the WNA and WSA. This latter assertion is based on the concordance of significant differentiation from both nuclear and mitochondrial markers between populations. Furthermore, it is also possible that the observed shallow differentiation between hemispheres was a result of a sampling artifact caused by assessing individuals from distinct populations, captured as a mixed assemblage of migratory adults. These equivocal results, especially placed in context of known overfishing of other billfish species - which is very likely also occurring for roundscale spearfish [18] - and the need for precautionary management principles for billfish in general [77], leads us to recommend that roundscale spearfish be recognized for future assessments and conservation on a two MU basis comprising northern and southern hemisphere stocks.

### Comparative mitochondrial DNA-based genetic diversity and demographic histories of the roundscale spearfish and white marlin

With one exception (see below), all statistical tests (Table 2 and Suppl. online Additional file 1) and Bayesian coalescent-based methods for inferring historical population trends were concordant in supporting strong signals of population expansion for both billfish species. However, the mismatch distributions for the roundscale spearfish and white marlin differed, being smooth and unimodal for the roundscale spearfish but ragged and multimodal for the white marlin (Suppl. online Additional file 2). The distribution curve for the roundscale spearfish was consistent with a demographic history of sudden expansion (or exponential growth [54]), but the distribution for white marlin was inconsistent with the expansion model. We note, however, that the white marlin mismatch distribution failed to statistically deviate from model expectations of expansion (see *Hri* and SSD in Suppl. Online Additional file 1). The reason for this discrepancy between the observed multimodal mismatch distribution curve and statistical fit is unclear. Collectively, however, the majority of the demographic results overwhelmingly support the scenario that both species have experienced substantial historical growth throughout the Pleistocene, consistent with findings for a number of other large pelagic species (e.g., [3,78,79]). Interestingly, the roundscale spearfish and white marlin mismatch distributions suggest that a population expansion began between ~200 000 – 600 000 ybp. This temporal window overlaps several Pleistocene interglacial periods, including one of the warmest and longest interglacials (the M11) which occurred approximately 400 000 ybp [80], which would have provided billfish with the opportunity for population expansion. However, we recognize that these estimates are based entirely on the assumed mutation rate, and may not accurately reflect the appropriate temporal window of population growth.

Estimates of roundscale spearfish nucleotide and haplotype diversity fell within those reported for the mtCR of other billfishes [23,43,81,82]. Interestingly, however, comparison of the genetic diversity indices of the roundscale spearfish and white marlin revealed estimates (nucleotide and Θ) to be consistently higher for white marlin, although substantial overlap of confidence intervals was present (Table 2). Overall, estimates of white marlin diversity (nucleotide and Θ) were 1.5-1.9 times those of the pooled collections of roundscale spearfish. Assuming equal mutation rates, generation times, and the selective neutrality of mtCR, these results suggests that the historical *Nf*_E_ of white marlin may be larger (1.5 to 1.9 times) than roundscale spearfish. However, it is important to note additional caveats related to this inference.

To date, no information is available on the generation time and growth of the roundscale spearfish [83], and small differences in generation time between the two species may lead to notable differences in estimates of their effective population size. Both species, however, occupy similar habitats and likely possess many similar life history characters, supporting the hypothesis of similar generation times.

Coalescent-based estimates of the female effective population size also suggested a higher effective size for white marlin. Final median estimates of *Nf*_E_ derived from the BSPs (effective female population size x generation time) for white marlin were approximately 1.5 to 1.9 times greater than for roundscale spearfish (Figure 3), although again substantial overlap of credibility intervals was present. Furthermore, LAMARC-derived [59] coalescent-based estimates of white marlin and roundscale spearfish *Nf*_E_ similarly showed a higher population size of white marlin relative to roundscale spearfish. These *Nf*_E_ estimates represent the long-term female effective population size of these two species (weighted harmonic mean over 2*N*_E_ generations), suggesting that historically white marlin population size has been larger than that of the roundscale spearfish.

As stated above, because the estimates of female effective population size generated herein are based on a single mitochondrial region, and the assumption of equal generation times and mutation rates for the two species, the absolute and even relative *Nf*_E_s should be considered highly provisional. Moreover, it is important to note that coalescent-based analyses incorporate numerous sources of error when estimating both the genealogy and population history from a small sample of individuals [84], and that similar population histories may generate highly variable BSPs (including variability in coalescent times) [85], suggesting that our BSPs and our relative estimates of population size should be interpreted with caution. Interestingly, however, although the higher *Nf*_E_s for white marlin compared to roundscale are preliminary, they are concordant with recent species observations of catch proportion data from fisheries which suggest that white marlin census population size is higher than that of roundscale spearfish, at least in the WNA and Caribbean waters [18,86,87]. Assuming an equal susceptibility of both billfish species to the fishery and a 1:1 sex ratio, the comparative *Nf*_E_ estimates suggest that white marlin census size may also be close to two times higher than that of roundscale spearfish. What are the implications of a roundscale spearfish population size that is much lower than the white marlin population size? It is well established that white marlin have been severely overfished [10] and that its population biomass may be as low as 12% of the biomass required to produce maximum sustainable yield. There are no data on roundscale spearfish landings to assess the status of its stocks, but it is very likely that is has historically been landed as “white marlin” because it’s taxonomic existence in fisheries data has been recognized only recently [11]. Since its recognition, three studies conducted in commercial and recreational fisheries have confirmed that the roundscale spearfish can make up a substantial proportion (~22-27%) of the putative “white marlin” catch [18,86,87].

To examine the effects of misidentification of roundscale spearfish on population trends of both roundscale spearfish and white marlin, Beerkircher et al. [18] conducted population assessment simulations under various demographic scenarios. The majority of simulation outcomes showed steeply declining population trends for both species, but even greater declines for roundscale spearfish relative to white marlin. With a much smaller apparent population size for roundscale spearfish, as suggested by the genetic analyses and fisheries data, continued exploitation of this species at current levels raises considerable concern about the long-term population health of this recently recognized species.

## Conclusions

The population structure and comparative demographics results presented here underscore the importance of changing from the current international management model for “white marlin” as a two species complex to management of the roundscale spearfish and white marlin as distinct evolutionary lineages. We recognize that a shift to species-specific management will be challenging due to misidentifications. However, there are now morphological identification tools available to distinguish the two species [12,17], and the increasing use of genetic tools can assist in this process.

Finally, we recognize that our conclusions pertaining to roundscale spearfish population structure and demographics are based on analyses of samples from only the western Atlantic, which may represent just part of this species’ potential Atlantic-wide distribution. However, our findings on these parameters placed in context of general billfish overfishing, almost no information on the life history of the roundscale spearfish, and the difficulty of enforcing pelagic fishery regulations on the international level, call for an aggressive precautionary management policy for this enigmatic species. Individual species management and conservation attention, including management on a two stock precautionary basis, are prudent to avoid inadvertent, drastic reductions in what appears to be a lower abundance species, and unrecognized population collapse of either potential roundscale spearfish hemispheric stock.

## Additional files

Additional file 1:
**Mitochondrial control region parameter estimates for mismatch distributions.** RS, roundscale spearfish; WM, white marlin; WNA, western North Atlantic; WSA, western South Atlantic; τ, tau derived from mismatch distribution; Θ_0_, theta at time 0 derived from mismatch distribution, Θ_1_, theta at time 1 derived from mismatch distribution; *Hr*, Harpending’s (1994) Raggedness index derived from mismatch distribution; SSD, sum of squared differences derived from mismatch distribution; *P*, probability. Additional file 2:
**Mismatch distributions for pooled samples of roundscale spearfish (RS:** ***Tetrapturus georgii*** **) and white marlin (WM:** ***Kajikia albida*** **).**
Additional file 3:
**Bayesian skyline plots (BSPs) for western North Atlantic (WNA) and western South Atlantic (WSA) roundscale spearfish (** ***Tetrapturus georgii*** **) populations.** BSPs derived using a mitochondrial control region mutation rate of (a) 1.8% per site per million years, and (b) 4.5% per site per million years.

## References

[1] Palumbi SR (1994). Genetic divergence, reproductive isolation and marine speciation. Annu Rev Ecol Syst.

[2] Waples RS (1998). Separating the wheat from the chaff: patterns of genetic differentiation in high gene flow species. J Hered.

[3] Theisen TC, Bowen BW, Lanier W, Baldwin JD (2008). High connectivity on a global scale in the pelagic wahoo, *Acanthocybium solandri* (tuna family Scombridae). Mol Ecol.

[4] Hauser L, Carvalho GR (2008). Paradigm shifts in marine fisheries genetics: ugly hypotheses slain by beautiful facts. Fish Fish.

[5] Riccioni G, Landi M, Ferrara G, Milano I, Cariani A, Zane L, Sella M, Barbujani G, Tinti F (2010). Spatio-temporal population structuring and genetic diversity retention in depleted Atlantic bluefin tuna of the Mediterranean Sea. Proc Natl Acad Sci U S A.

[6] Knutsen H, Olsen EM, Jorde PE, Espeland SH, André C, Stenseth NC (2011). Are low but statistically significant levels of genetic differentiation in marine fishes 'biologically meaningful'? A case study of coastal Atlantic cod. Mol Ecol.

[7] Anonymous (2011). Report of the 2011 blue marlin stock assessment and white marlin data preparatory meeting, Madrid, Spain April 25–29, 2011. Coll Vol Sci Papers ICCAT.

[8] Collette B, Amorim AF, Bizsel K, Boustany A, Carpenter KE, de Oliveira Leite Jr N, Die D, Fox W, Fredou FL, Graves J, Viera Hazin FH, Hinton M, Juan Jorda M, Masuti E, Minte Vera C, Miyabe N, Nelson R, Oxenford H, Restrepo V, Schratwieser J, Teixeira Lessa RP, Pires Ferreira Travassos PE: *Kajikia albida*: IUCN Red List of Threatened Species. Version 2013.2. [http://www.iucnredlist.org/details/170322/0]

[9] Collette BB, Carpenter KE, Polidoro BA, Juan-Jordá MJ, Boustany AM, Die DJ, Elfes C, Fox W, Graves J, Harrison LR, McManus R, Minte-Vera CV, Nelson R, Restrepo V, Schratwieser J, Sun C-L, Amorim A, Brick Peres M, Canales C, Cardenas G, Chang S-K, Chiang W-C, De Oliveira Leite Jr N, Harwell H, Lessa R, Fredou FL, Oxenford HA, Serra R, Shao K-T, Sumaila R (2011). High value and long life - double jeopardy for tunas and billfishes. Science.

[10] ICCAT (International Commission for the Conservation of Atlantic Tunas) (2013). ICCAT Report for the biennial period, 2012 – 13, Part I – volume 2, Madrid, Spain. ICCAT.

[11] Shivji M, Magnussen JE, Beerkircher LR, Hinteregger GF, Lee DW, Serafy JE, Prince ED (2006). Validity, identification, and distribution of the roundscale spearfish, *Tetrapturus georgii* (Teleostei: Istiophoridae): morphological and molecular evidence. Bull Mar Sci.

[12] Beerkircher LR, Lee DW, Hinteregger GF (2008). Roundscale spearfish *Tetrapturus georgii*: morphology, distribution, and relative abundance in the western north Atlantic. Bull Mar Sci.

[13] Bernard AM, Shivji MS, Domingues RR, Viera Hazin FH, De Amorim AF, Domingo A, Arocha F, Prince ED, Hoolihan JP, Hilsdorf AWS (2013). Broad geographic distribution of roundscale spearfish (*Tetrapturus georgii*) (Teleostei, Istiophoridae) in the Atlantic revealed by DNA analysis: implications for white marlin and roundscale spearfish management. Fish Res.

[14] Schrope M (2013). Fishy numbers for white marlin stocks. Proc Natl Acad Sci U S A.

[15] Collette BB, McDowell JR, Graves JE (2006). Phylogeny of recent billfishes (Xiphioidei). Bull Mar Sci.

[16] Hanner R, Floyd R, Bernard A, Collette BB, Shivji M (2011). DNA barcoding in billfishes. Mitochrondr DNA.

[17] Beerkircher LR, Serafy JE (2011). Using head measurements to distinguish white marlin (*Tetrapturus albidus*) from roundscale spearfish (*T. georgii*). Bull Mar Sci.

[18] Beerkircher LR, Arocha F, Barse A, Prince ED, Restrepo V, Serafy JE, Shivji MS (2009). Effects of species misidentification on population assessment of overfished white marlin *Tetrapturus albidus* and roundscale spearfish *T. georgii*. Endanger Species Res.

[19] WMBRT (White Marlin Billfish Review Team) (2007). Atlantic White Marlin Status Review.

[20] Arocha F, Bárrios A (2009). Sex ratios, spawning seasonality, sexual maturity, and fecundity of white marlin (*Tetrapturus albidus*) from the western central Atlantic. Fish Res.

[21] Rocha LA (2003). Patterns of distribution and processes of speciation in Brazilian reef fishes. J Biogeogr.

[22] Rocha LA, Craig MT, Bowen BW (2007). Phylogeography and the conservation of coral reef fishes. Coral Reefs.

[23] Graves JE, McDowell JR (2006). Genetic analysis of white marlin (*Tetrapturus albidus*) stock structure. Bull Mar Sci.

[24] Alvarado Bremer JR, Mejuto J, Gómez-Márquez J, Boán F, Carpintero P, Rodríguez JM, Viñas J, Greig TW, Ely B (2005). Hierarchical analyses of genetic variation of samples from breeding and feeding grounds confirms the genetic partitioning of northwest Atlantic and South Atlantic populations of swordfish (*Xiphias gladius* L.). J Exp Mar Biol Ecol.

[25] Bernard AM, Feldheim KA, Shivji MS (2012). Development and characterization of 11 novel microsatellite loci for the roundscale spearfish *Tetrapturus georgii* and their cross-species amplifcation among other istiophorid species. J Fish Biol.

[26] Buonaccorsi VP, Graves JE (2000). Isolation and characterization of novel polymorphic tetra-nucleotide microsatellite markers from the blue marlin, *Makaira nigricans*. Mol Ecol.

[27] Schuelke M (2000). An economic method for the fluorescent labeling of PCR fragments. Nat Biotechnol.

[28] Raymond M, Rousset F (1995). GENEPOP (version 1.2): population genetics software for exact tests and ecumenicism. J Hered.

[29] Rousset F (2008). Genepop '007: a complete reimplementation of the GENEPOP software for Windows and Linux. Mol Ecol Resour.

[30] Guo SW, Thompson EA (1992). Performing the exact test of Hardy-Weinberg proportion for multiple alleles. Biometrics.

[31] Rousset F, Raymond M (1995). Testing hertozygote excess and deficiency. Genetics.

[32] Rice WR (1989). Analyzing tables of statistical tests. Evolution.

[33] El Mousadik A, Petit RJ (1996). High level of genetic differentiation for allelic richness among populations of the argan tree (*Argania spinosa* (L.) Skeels) endemic to Morocco. Theor Appl Genet.

[34] Goudet J: FSTAT, a program to estimate and test gene diversities and fixation indices v2.9.3 Release [http://www2.unil.ch/popgen/softwares/fstat.htm]

[35] Chapuis M-P, Estoup A (2007). Microsatellite null alleles and estimation of population differentiation. Mol Biol Evol.

[36] Excoffier L, Laval G, Schneider S (2005). Arlequin (version 3.0): an integrated software package for population genetics data analysis. Evol Bioinf Online.

[37] Jost L: ***G***_ST_**and its relatives do not measure differentiation.** *Mol Ecol* 2008, **17:**4015–4026. 10.1111/j.1365-294x.2008.03887.x 19238703

[38] Chao A, Shen T-J: Program SPADE (Species Prediction And Diversity Estimation) [http://chao.stat.nthu.edu.tw/blog/software-download/spade/]

[39] Hudson RR (2000). A new statistic for detecting genetic differentiation. Genetics.

[40] Librado P, Rozas J (2009). DnaSP v5: a software for comprehensive analysis of DNA polymorphism data. Bioinformatics.

[41] Gerlach G, Jueterbock A, Kraemer P, Deppermann J, Harmand P: **Calculations of population differentiation based on G**_ST_**and D: forget G**_ST_**but not all of statistics.** *Mol Ecol* 2010, **19:**3845–3852. 10.1111/j.1365-294X.2010.04784.x 20735737

[42] R Development Core Team (2012). R: A Language and Environment for Statistical Computing.

[43] Muths D, Grewe P, Jean C, Bourjea J (2009). Genetic population structure of the Swordfish (*Xiphias gladius*) in the southwest Indian Ocean: Sex-biased differentiation, congruency between markers and its incidence in a way of stock assessment. Fish Res.

[44] Pritchard JK, Stephens M, Donnelly P (2000). Inference of population structure using multilocus genotype data. Genetics.

[45] Falush D, Stephens M, Pritchard JK (2003). Inference of population structure: extensions to linked loci and correlated allele frequencies. Genetics.

[46] Hubisz MJ, Falush D, Stephens M, Pritchard JK (2009). Inferring weak population structure with the assistance of sample group information. Mol Ecol Resour.

[47] Guillot G, Mortier F, Estoup A (2005). Geneland: a computer package for landscape genetics. Mol Ecol Notes.

[48] Peakall R, Smouse PE (2006). GenAlEx 6: genetic analysis in Excel. Population genetic software for teaching and research. Mol Ecol Notes.

[49] Guindon S, Gascuel O (2003). A simple, fast and accurate method to estimate large phylogenies by maximum-likelihood. Syst Biol.

[50] Posada D (2008). jModelTest: phylogenetic model averaging. Mol Biol Evol.

[51] Nei M (1987). Molecular Evolutionary Genetics.

[52] Fu YX (1996). Estimating the age of the common ancestor of a DNA sample using the number of segregating sites. Genetics.

[53] Ramos-Onsins SE, Rozas J (2002). Statistical properties of new neutrality tests against population growth. Mol Biol Evol.

[54] Rogers AR, Harpending H (1992). Population growth makes waves in the distribution of pairwise genetic differences. Mol Biol Evol.

[55] Harpending HC (1994). Signature of ancient population growth in a low-resolution mitochondrial DNA mismatch distribution. Hum Biol.

[56] Donaldson KA, Wilson RRJ (1999). Amphi-Panamic germinates of snook (Percoidei: Centropomidae) provide a calibration of the divergence rate in the mitochondrial DNA control region of fishes. Mol Phylogenet Evol.

[57] Sato A, Takezaki N, Tichy H, Figueroa F, Mayer WE, Klein J (2003). Origin and speciation of haplochromine fishes in east African crater lakes investigated by the analysis of their mtDNA, *Mhc* genes, and SINEs. Mol Biol Evol.

[58] Bowen BW, Muss A, Rocha LA, Grant WS (2006). Shallow mtDNA coalescence in Atlantic pygmy angelfishes (Genus *Centropyge*) indicates a recent invasion from the Indian Ocean. J Hered.

[59] Kuhner MK (2006). Lamarc 2.0: maximum likelihood and Bayesian estimation of population parameters. Bioinformatics.

[60] Kuhner MK (2009). Coalescent genealogy samplers: windows into population history. Trends Ecol Evol.

[61] Rambaut A, Drummond AJ: Tracer v1.5. [http://beast.bio.ed.ac.uk/Tracer]

[62] Drummond AJ, Rambaut A (2007). BEAST: Bayesian evolutionary analysis by sampling trees. BMC Evol Biol.

[63] van Oosterhout C, Hutchinson WF, Wills DPM, Shipley PF: Micro-Checker User Guide v2.3 [http://www.microchecker.hull.ac.uk/]

[64] Hasegawa M, Kishino K, Yano T (1985). Dating the human-ape splitting by a molecular clock of mitochondrial DNA. J Mol Evol.

[65] Ryman N, Palm S, André C, Carvalho GR, Dahlgren TG, Jorde PE, Laikre L, Larsson LC, Palmé A, Ruzzante DE (2006). Power for detecting genetic divergence: differences between statistical methods and marker loci. Mol Ecol.

[66] Waples RS, Gaggiotti O (2006). What is a population? An empirical evaluation of some genetic methods for identifying the number of gene pools and their degree of connectivity. Mol Ecol.

[67] Latch EK, Dharmarajan G, Glaubitz JC, Rhodes OEJ (2006). Relative performace of Bayesian clustering software for inferring population substructure and individual assignment at low levels of population differentiation. Conserv Genet.

[68] Orozco-terWengel P, Corander J, Schlötterer C (2011). Genealogical lineage sorting leads to significant, but incorrect Bayesian multilocus inference of population structure. Mol Ecol.

[69] Balloux F, Lugon-Moulin N (2002). The estimation of population differentiation with microsatellite markers. Mol Ecol.

[70] Ryman N, Leimar O: ***G***_ST_**is still a useful measure of genetic differentation - a comment on Jost's*****D*****.** *Mol Ecol* 2009, **18:**2084–2087. 10.1111/j.1365-294X.2009.04187.x 19389168

[71] Buonaccorsi VP, Reece KS, Morgan LW, Graves JE (1999). Geographic distribution of molecular variance within the blue marlin (*Makaira nigricans*): a hierarchical analysis of allozyme, single-copy nuclear DNA, and mitochondrial DNA markers. Evolution.

[72] Buonaccorsi VP, McDowell JR, Graves JE (2001). Reconciling patterns of inter-ocean molecular variance from four classes of molecular markers in blue marlin (*Makaira nigricans*). Mol Ecol.

[73] Graves JE, McDowell JR (2003). Stock structure of the world’s istiophorid billfishes: a genetic perspective. Mar Freshwater Res.

[74] McDowell JR, Carlsson J, Graves JE (2007). Genetic analysis of blue marlin (*Makaira nigricans*) stock structure in the Atlantic ocean. Gulf Caribb Res.

[75] Dutton PH, Roden SE, Stewart KR, LaCasella E, Tiwari M, Formia A, Thomé JC, Livingstone SR, Eckert S, Chacon-Chaverri D, Rivalan P, Allman P (2013). Population stock structure of leatherback turtles (*Dermochelys coriacea*) in the Atlantic revealed using mtDNA and microsatellite markers. Conserv Genet.

[76] Moritz C (1994). Defining ‘Evolutionary Significant Units’ for conservation. Trends Ecol Evol.

[77] Die DJ (2006). Are Atlantic marlins overfished or endangered? Some reasons why we may not be able to tell. Bull Mar Sci.

[78] Carlsson J, McDowell JR, Díaz-Jaimes P, Carlsson JEL, Boles SB, Gold JR, Graves JE (2004). Microsatellite and mitochondrial DNA analyses of Atlantic bluefin tuna (*Thunnus thynnus thynnus*) population structure in the Mediterranean Sea. Mol Ecol.

[79] Alvarado Bremer JR, Viñas J, Mejuto J, Ely B, Pla C (2005). Comparative phylogeography of Atlantic bluefin tuna and swordfish: the combined effects of vicariance, secondary contact, introgression, and population expansion on the regional phylogenies of two highly migratory pelagic fishes. Mol Phylogenet Evol.

[80] Droxler AW, Alley RB, Howard WR, Poore RZ, Burckle LH (2003). Unique and exceptionally long interglacial marine isotope stage 11: window into earth warm future climate. Geoph Monog Series.

[81] McDowell JR, Graves JE (2008). Population structure of striped marlin (*Kajikia audax*) in the Pacific Ocean based on analysis of microsatellite and mitochondrial DNA. Can J Fish Aquat Sci.

[82] Purcell CM, Edmands S (2011). Resolving the genetic structure of striped marlin, *Kajikia audax*, in the Pacific Ocean through spatial and temporal sampling of adult and immature fish. Can J Fish Aquat Sci.

[83] Hoolihan J: ICCAT Manual Chapter 2.1.8.4. Roundscale Spearfish [http://www.iccat.int/Documents/SCRS/Manual/CH2/2_1_8_4_SPG_ENG.pdf]

[84] Ho SYW, Shapiro B (2011). Skyline-plot methods for estimating demographic history from nucleotide sequences. Mol Ecol Resour.

[85] Karl SA, Toonen RJ, Grant WS, Bowen BW (2012). Common misconceptions in molecular ecology: echoes of the modern synthesis. Mol Ecol.

[86] Arocha F, Silva J (2011). Proportion of *Tetrapturus georgii* (SPG) with respect to *T. albidus* (WHM) in the Venezuelan pelagic longline catch in the western Caribbean Sea and adjacent Atlantic waters during 2002–2007. Coll Vol Sci Papers ICCAT.

[87] Graves JE, McDowell JR (2012). Inter-annual variability in the proportion of roundscale spearfish (*Tetrapturus georgii*) and and white marlin (*Kajikia albida*) in the western North Atlantic Ocean. Coll Vol Sci Papers ICCAT.

