# Supplementary material for: Comparative population genetics and evolutionary history of two commonly misidentified billfishes of management and conservation concern
Source: BMC Genet. 2014 Dec 14;15:141. doi: 10.1186/s12863-014-0141-4 (PMC4278234; doi:10.1186/s12863-014-0141-4)
Supplement: Additional file 1: — Mitochondrial control region parameter estimates for mismatch distributions. RS, roundscale spearfish; WM, white marlin; WNA, western North Atlantic; WSA, western South Atlantic; τ, tau derived from mismatch distribution; Θ0, theta at time 0 derived from mismatch distribution, Θ1, theta at time 1 derived from mismatch distribution; Hr, Harpending’s (1994) Raggedness index derived from mismatch distribution; SSD, sum of squared differences derived from mismatch distribution; P, probability. [file 12863_2014_141_MOESM1_ESM.docx]

**Supplementary online Additional File 1.** Mitochondrial control region parameter estimates for mismatch distributions. RS, roundscale spearfish; WM, white marlin; WNA, western North Atlantic; WSA, western South Atlantic; τ, tau derived from mismatch distribution; Θ_0_, theta at time 0 derived from mismatch distribution, Θ_1_, theta at time 1 derived from mismatch distribution; *Hr*, Harpending’s (1994) Raggedness index derived from mismatch distribution; SSD, sum of squared differences derived from mismatch distribution; *P*, probability.

| **Species and Sample** | **τ (95% CI)** | **Θ_0_** | **Θ_1_** | ***Hr*** | ***P*(*Hr*)** | **SD** | ***P*(SSD)** |
| --- | --- | --- | --- | --- | --- | --- | --- |
| RS WNA | 14.80  (9.36, 18.50) | 0.00 | 58.36 | 0.0057 | 0.72 | 0.0044 | 0.47 |
| RS WSA | 9.89  (5.46, 25.82) | 6.47 | 74.37 | 0.0038 | 0.88 | 0.0021 | 0.84 |
| RS overall | 10.97  (6.75, 23.76) | 4.82 | 88.44 | 0.0016 | 0.98 | 0.0008 | 0.87 |
| WM overall | 12.66  (8.09, 33.99) | 15.31 | 316.41 | 0.0009 | 0.94 | 0.0032 | 0.53 |
