# Supplementary material for: Comparative population genetics and evolutionary history of two commonly misidentified billfishes of management and conservation concern
Source: BMC Genet. 2014 Dec 14;15:141. doi: 10.1186/s12863-014-0141-4 (PMC4278234; doi:10.1186/s12863-014-0141-4)
Supplement: Additional file 2: — Mismatch distributions for pooled samples of roundscale spearfish (RS: Tetrapturus georgii ) and white marlin (WM: Kajikia albida ). [file 12863_2014_141_MOESM2_ESM.docx]

**Supplementary online Additional File 2.** Mismatch distributions for pooled samples of roundscale spearfish (RS: *Tetrapturus georgii*) and white marlin (WM: *Kajikia albida*).
