# Supplementary material for: Comparative population genetics and evolutionary history of two commonly misidentified billfishes of management and conservation concern
Source: BMC Genet. 2014 Dec 14;15:141. doi: 10.1186/s12863-014-0141-4 (PMC4278234; doi:10.1186/s12863-014-0141-4)
Supplement: Additional file 3: — Bayesian skyline plots (BSPs) for western North Atlantic (WNA) and western South Atlantic (WSA) roundscale spearfish ( Tetrapturus georgii ) populations. BSPs derived using a mitochondrial control region mutation rate of (a) 1.8% per site per million years, and (b) 4.5% per site per million years. [file 12863_2014_141_MOESM3_ESM.docx]

(a)

(b)

**Supplementary online Additional File 3.** Bayesian skyline plots (BSPs) for western North Atlantic (WNA) and western South Atlantic (WSA) roundscale spearfish (*Tetrapturus georgii*) populations. BSPs derived using a mitochondrial control region mutation rate of (a) 1.8% per site per million years, and (b) 4.5% per site per million years.
